# Assessing the Effect of Sodium-Glucose Cotransporter 2 Inhibitor (SGLT2i) on Outcomes in Patients With Acute Myocardial Infarction: A Systematic Review and Meta-Analysis

**DOI:** 10.7759/cureus.62978

**Published:** 2024-06-23

**Authors:** Scott Nall, Anurag Rawat, Fahad Shaukat Gill, Rushna Saleem, Simran Saeed, Saeed Ahmed, Calvin R Wei, Danish Allahwala

**Affiliations:** 1 Medicine, Central Michigan University College of Medicine, Saginaw, USA; 2 Interventional Cardiology, Himalayan Institute of Medical Sciences, Dehradun, IND; 3 Medicine, Shalamar Medical and Dental College, Lahore, PAK; 4 Medicine, Rawalpindi Medical University, Rawalpindi, PAK; 5 Allied Health, University of Lahore, Lahore, PAK; 6 Cardiology, Mohtarma Benazir Bhutto Shaheed Medical College, Mirpur, PAK; 7 Research and Development, Shing Huei Group, Taipei, TWN; 8 Nephrology, Fatima Memorial Hospital, Karachi, PAK

**Keywords:** systematic review and meta-analysis, mortality, cardiovascular outcomes, myocardial infarction, sodium glucose co-transporter 2 inhibitors (sglt2i)

## Abstract

After acute myocardial infarction, patients are at increased risk for adverse outcomes, including heart failure and death. Sodium-glucose cotransporter 2 inhibitors (SGLT2i) have shown promising cardiovascular benefits, but their efficacy in patients after myocardial infarction is not well established. This study aimed to evaluate the efficacy of SGLT2i in preventing cardiovascular outcomes in patients after myocardial infarction through a systematic review and meta-analysis. We conducted a comprehensive literature search of PubMed, Cochrane, EMBASE, and Web of Science for randomized controlled trials (RCTs) and retrospective and prospective studies evaluating SGLT2i in patients after myocardial infarction. The primary outcomes were major adverse cardiovascular events (MACEs) and all-cause mortality. Secondary outcomes included cardiovascular mortality, recurrent myocardial infarction, revascularization, and rehospitalization. Data were pooled using a random-effects model, and risk ratios (RRs) with 95% confidence intervals (CIs) were calculated. The meta-analysis included eight studies (three RCTs and five observational studies) with a follow-up duration ranging from 4 to 24 months. SGLT2i were associated with a significantly lower risk of MACE (RR: 0.71, 95% CI: 0.52-0.97, p = 0.03) and rehospitalization (RR: 0.64, 95% CI: 0.51-0.82, p<0.01) compared to controls. Although not statistically significant, the risk of all-cause mortality (RR: 0.79, 95% CI: 0.53-1.18, p = 0.25) and cardiovascular mortality was lower in the SGLT2i group. This meta-analysis suggests that SGLT2i may improve cardiovascular outcomes in patients after myocardial infarction, particularly by reducing the risk of MACEs and rehospitalization. However, larger trials with high-risk populations are needed to confirm these findings and elucidate the underlying mechanisms.

## Introduction and background

After acute myocardial infarction, patients are at an increased risk for heart failure and death, particularly if they present with congestion or a decreased left ventricular ejection fraction [1]. A complicated mechanism underlies the pathophysiology of myocardial infarction. It includes energy-depleting acute myocardial ischemia, early reperfusion injury that develops in the first few minutes or hours of reperfusion, and the remodeling phase that occurs in the early days or weeks following myocardial infarction, which results in irreversible necrotic damage to the area of concern [2]. Treatment with sodium-glucose cotransporter 2 inhibitors (SGLT2i) improves cardiovascular outcomes in high-risk patients with type 2 diabetes, those with chronic kidney disease, and those with heart failure with a reduced or preserved left ventricular ejection fraction [3].

Starting and maintaining SGLT2i early for acute myocardial infarction is promising due to several suggested mechanisms that could change the natural course of the condition, reduce the likelihood of ventricular remodeling, and slow the progression to chronic heart failure and end-stage heart disease [4]. Recent findings from experimental acute myocardial infarction models in both diabetic and non-diabetic subjects have demonstrated numerous benefits from SGLT2 inhibition [5-6]. Remarkably, the beneficial cardiovascular effects observed in cardiovascular outcome trials (CVOTs) appeared within just a few weeks of starting treatment and were found to be independent of glycemic status [7-8].

Due to the increasing evidence in various disease states and the suggested mechanisms of action, it seems reasonable to explore the potential of SGLT2 inhibition in improving outcomes for patients with acute myocardial infarction if administered promptly after the presentation. However, the safety and effectiveness of these therapies after myocardial infarction are not well understood. Therefore, the present study aims to use current literature and conduct a pooled analysis to determine the efficacy of SGLT2i in preventing cardiovascular outcomes in patients after myocardial infarction.

## Review

Methodology

We conducted this study according to the Preferred Reporting Items for Systematic Reviews and Meta-analyses (PRISMA) guidelines.

Search Strategy and Study Selection

We carried out a comprehensive search in PubMed, Cochrane, EMBASE, and Web of Science to analyze the effectiveness of SGLT2i on cardiovascular outcomes in patients after myocardial infarction compared to placebo or other drugs using the following terms along with their synonyms and medical subject heading (MeSH) terms: “SGLT2i," “myocardial infarction," “all-cause mortality,” and “cardiovascular outcomes” from inception to May 15, 2024. Relevant randomized controlled trials (RCTs) or any retrospective or prospective study that focused on objectives were included. We included studies that were exclusively conducted in patients with myocardial infarction. We excluded animal studies, reviews, and editorials. If a study was a subanalysis of an RCT, we considered the primary RCT to avoid data duplication. However, if a subanalysis offered additional information not included in the primary RCT, we included that data for analyzing specific parameters. Similarly, if a study reported values for both intention to treat (ITT) and per protocol analysis (PPA), we used the ITT values to minimize bias. Screening was performed by two authors independently. In the first phase, abstract and title screening were done followed by detailed assessment based on predefined inclusion and exclusion criteria.

Data Collection and Quality Assessment

The included studies were evaluated to summarize the author’s name, publication year, study design, number of participants, type of SGLT2i, follow-up duration, and outcomes assessed in this meta-analysis. Primary outcomes assessed in this study included major adverse cardiovascular events (MACEs) and all-cause mortality. Secondary outcomes included cardiovascular mortality, myocardial infarction, and rehospitalization. Data were extracted by one author and were cross-checked by the second one. Any disagreement between two authors was resolved through discussion. Quality assessment was performed by two authors independently using the Cochrane Risk-of-Bias Assessment tool for RCT and the Newcastle-Ottawa Scale for observational studies. Any disagreement between two authors was resolved through discussion.

Statistical Analysis 

The meta-analysis was conducted using Review Manager (RevMan) version 5.4 (The Cochrane Collaboration). A random-effects model was chosen over a fixed-effects model to mitigate the potential impact of interstudy variability on the effect estimate. The outcomes between the two groups were compared using the risk ratio (RR) with a 95% confidence interval (CI). A P-value of less than 0.05 was deemed statistically significant. Statistical heterogeneity (I²), which indicates the degree of variation among the included studies, was assessed to evaluate heterogeneity. An I^2^ statistic greater than 50% was considered indicative of significant heterogeneity. 

Results

**Figure 1:**
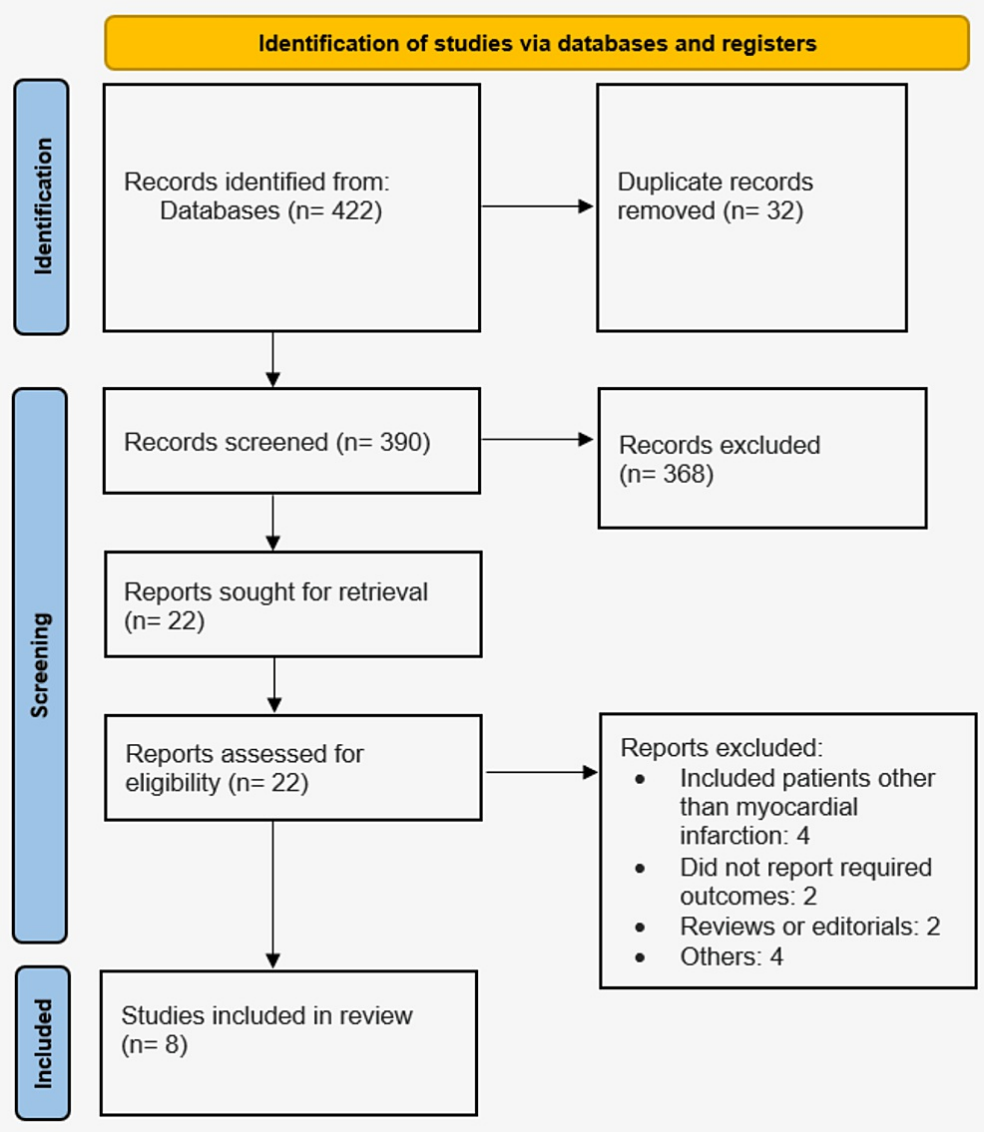
Preferred Reporting Items for Systematic Reviews and Meta-analyses (PRISMA) flowchart of the study selection

In the initial screening, 422 articles were retrieved from the database search. After eliminating 32 duplicate studies, 390 articles remained for the preliminary review. From these, 22 studies underwent a detailed evaluation based on predefined inclusion and exclusion criteria. Ultimately, eight studies were included in this meta-analysis, comprising three RCTs and five observational studies. Figure 1 shows the process of study selection. Table 1 presents the characteristics of the included studies. The follow-up duration for the included studies ranged from four to 24 months. Table 2 presents the quality assessment of the included studies. 

**Table 1:** Characteristics of the included studies SGLT2i: sodium-glucose co-transporter 2 inhibitor; RCT: randomized controlled trial; NS: not specified

Author	Year	Study design	Region	Groups	Population	Type of SGLT2i	Follow-up duration
Butler et al. [9]	2024	RCT	Multi-national	SGLT2i	3260	Empagliflozin	17.9 months
				Non-SGLT2i	3262		
Chang et al. [10]	2022	Observational	Taiwan	SGLT2i	66	Dapagliflozin or Empagliflozin	23.5 months
				Non-SGLT2i	132		
James et al. [11]	2024	RCT	Multi-national	SGLT2i	2019	Dapagliflozin	24 months
				Non-SGLT2i	1998		
Kwon et al. [12]	2023	Observational	Korea	SGLT2i	938	Dapagliflozin or Empagliflozin	24 months
				Non-SGLT2i	1876		
Lewinski et al. [13]	2022	RCT	Austria	SGLT2i	237	Empagliflozin	4 months
				Non-SGLT2i	239		
Lyu et al. [14]	2023	Observational	Korea	SGLT2i	186	NS	12 months
				Non-SGLT2i	593		
Mao et al. [15]	2023	Observational	China	SGLT2i	275	Dapagliflozin	18 months
				Non-SGLT2i	686		
Paolisso et al. [16]	2023	Observational	Belgium	SGLT2i	111	NS	24 months
				Non-SGLT2i	535		

**Table 2:** Risk-of-bias assessment

Author	Selection	Comparison	Assessment	Overall	
Quality assessment of observational studies					
Chang et al. [10]	4	2	2	Good	
Kwon et al. [12]	3	2	2	Good	
Lyu et al. [14]	4	2	3	Good	
Mao et al. [15]	4	1	2	Good	
Paolisso et al. [16]	3	2	3	Good	
Quality assessment of randomized control trials					
Author	Randomization	Allocation concealment	Blinding	Selective reporting	Other bias
Butler et al. [9]	Low	Low	Low	Low	Low
James et al. [11]	Low	Unclear	Low	Low	Low
Lewinski et al. [13]	Low	Low	Unclear	Low	Low

Meta-Analysis of Outcomes

**Figure 2:**
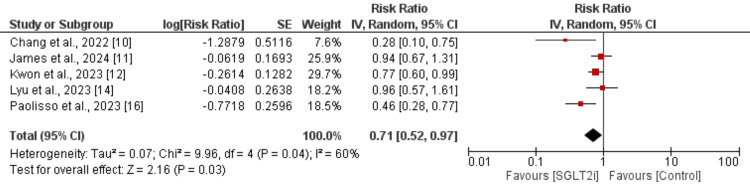
Effect of SGLT2i on MACEs in myocardial infarction patients SGLT2i: sodium-glucose co-transporter 2 inhibitor, MACE: major adverse cardiovascular event Sources: References [10-12,14,16]

**Figure 3:**
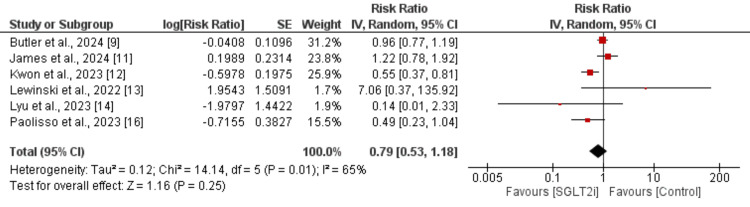
Effect of SGLT2i on all-cause death in myocardial infarction patients SGLT2i: sodium-glucose co-transporter 2 inhibitor Sources: References [9,11-14,16]

Primary outcomes (MACEs and all-cause mortality): We compared MACEs and all-cause mortality between the two study groups as primary outcomes, with the results shown in Figure 2 and Figure 3, respectively. The pooled analysis of MACEs included five studies (four observational studies and one RCT). This analysis indicated that the risk of developing MACEs was significantly lower in patients receiving SGLT2i compared to the control group (RR: 0.71, 95% CI: 0.52 to 0.97, p-value = 0.03), with a significant heterogeneity reported (I²: 60%).

For the pooled analysis of all-cause mortality, six studies were included (three RCTs and three observational studies). As shown in Figure 3, the risk of all-cause mortality was lower in the SGLT2i group, but the difference between the two groups was not significant (RR: 0.79, 95% CI: 0.53 to 1.18, p-value = 0.25). We performed a subgroup analysis of all-cause mortality based on the study design. This analysis revealed a significant difference between the results of RCTs and observational studies. The pooled analysis of observational studies showed that the use of SGLT2i is associated with a reduced risk of all-cause mortality (RR: 0.53, 95% CI: 0.37 to 0.74, I^2^: 0%). However, the pooled analysis of RCTs did not show any significant difference between the two groups (RR: 1.05, 95% CI: 0.79 to 1.39, I^2^: 22%). 

Secondary outcomes: The findings of the pooled analysis of secondary outcomes including cardiovascular death, recurrent myocardial infarction, and rehospitalization are shown in Table 3. The pooled analysis showed that the risk of cardiovascular death is not significantly different between patients in the SGLT2i and control groups. Similarly, the risk of developing recurrent myocardial infarction is also not significant between the two study groups. On the other hand, the SGLT2i is significantly associated with a lower risk of hospitalization due to cardiovascular reasons (RR: 0.64, 95% CI: 0.51 to 0.82, p-value < 0.01). 

**Table 3:** Analysis of the secondary outcomes RR: risk ratio; CI: confidence interval

Outcome	RR (95% CI)	I^2^
Cardiac death	0.90 (0.60 to 1.34)	29%
Myocardial infarction	1.03 (0.79 to 1.33)	0%
Rehospitalization	0.64 (0.51 to 0.82)	38%

Discussion

Patients who have experienced a myocardial infarction are at risk for adverse outcomes, including recurrent myocardial infarction, chronic heart failure, life-threatening arrhythmias, and cardiovascular death [17]. This updated meta-analysis incorporates two recently conducted RCTs that evaluated the effects of SGLT2i on outcomes in post-myocardial infarction patients. Our findings indicate that SGLT2i are significantly associated with a reduced risk of MACE and rehospitalization. Although no significant differences were observed between the two groups regarding all-cause mortality and cardiovascular mortality, the risk was lower in patients receiving SGLT2i. A recent meta-analysis by Sinha et al. [18] reported a significant reduction in the risk of MACE, all-cause mortality, cardiovascular mortality, and cardiovascular-related hospitalizations in patients treated with SGLT2i compared to those in the control group. However, that meta-analysis included studies involving general acute coronary syndrome patients. By contrast, our meta-analysis focused exclusively on studies conducted in patients with myocardial infarction.

The precise mechanisms underlying the beneficial effects of SGLT2i in these patient populations have not been fully elucidated. These benefits do not appear to be primarily related to glucose control but rather seem to result from direct cardioprotective and nephroprotective actions. Potential mechanisms include regulation of sodium balance, maintenance of energy homeostasis, reduction of cellular stress, enhancement of endothelial function, and promotion of vasodilation [19-20]. Animal studies have shown that SGLT2i can reduce mortality rates following myocardial infarction by altering cardiac metabolomes and increasing antioxidant levels in diabetic rats [21]. In addition, SGLT2i seem to reduce the size of myocardial infarctions, improve left ventricular (LV) function, and decrease the incidence of arrhythmias [22], collectively contributing to better cardiac outcomes. 

Previous reviews have indicated that the use of SGLT2i is significantly associated with a lower risk of all-cause mortality and cardiac-related mortality [23-24]. This meta-analysis includes two recently conducted RCTs. Neither of these RCTs reported a significant difference between the two groups regarding mortality risk. In the DAPA-MI trial, the actual number of deaths was too low to draw any meaningful conclusions [11]. In the EMPACT-MI trial [9], some patients with lower left ventricular ejection fractions or congestion at randomization may have had a stunned myocardium that was reversible; further improvement after revascularization is unlikely in this lower-risk population [25]. This might be particularly true for patients with ST-elevation myocardial infarction (STEMI), who made up nearly 75% of the EMPACT-MI trial participants, of whom approximately 90% underwent early revascularization. Despite these two trials not showing a significant prognostic benefit for SGLT2i, they have certain limitations. Therefore, future studies with high-risk populations and larger sample sizes are needed to accurately assess the effect of SGLT2i in these patients.

This meta-analysis has several limitations. First, only three clinical trials were included, with certain outcomes like MACEs reported by only one study. Therefore, future large-scale studies are needed to assess the effect of SGLT2i in preventing poor cardiovascular outcomes post-myocardial infarction. Second, the dose and duration of SGLT2i administration varied across the included studies, which may have introduced confounding bias. A significant limitation of this analysis is the inclusion of observational studies with low sample sizes, which contribute minimally to the overall pooling of results. This limitation undermines the robustness and generalizability of the findings. In addition, many results from the RCTs included in this review are not statistically significant, further questioning the strength of the evidence provided. Furthermore, we did not perform a publication bias assessment, such as a funnel plot or Egger's test, due to the small number of included studies. A reliable assessment of publication bias requires a larger number of studies to detect asymmetry accurately, and our dataset did not meet this requirement. Consequently, readers should interpret the findings with caution, considering these potential sources of bias and the overall limited evidential power.

## Conclusions

This meta-analysis suggests that SGLT2i may reduce the risk of MACEs and rehospitalization in patients after myocardial infarction. Although the impact on mortality was not statistically significant, the risk was lower in the SGLT2i group. These findings highlight the potential benefits of SGLT2i in improving outcomes for post-myocardial infarction patients. However, larger RCTs with high-risk populations are needed to confirm these findings and better understand the underlying mechanisms. Overall, this meta-analysis supports the consideration of SGLT2i as a therapeutic option for patients after myocardial infarction.

## References

[1] Harrington J, Jones WS, Udell JA (2022). Acute decompensated heart failure in the setting of acute coronary syndrome. JACC Heart Fail.

[2] Benedikt M, Kolesnik E, Sourij H, von Lewinski D (2023). SGLT2 inhibition in acute myocardial infarction—a comprehensive review. Rev Cardiovasc Med.

[3] Usman MS, Siddiqi TJ, Anker SD (2023). Effect of SGLT2 inhibitors on cardiovascular outcomes across various patient populations. J Am Coll Cardiol.

[4] Verma S, Anker SD, Butler J, Bhatt DL (2020). Early initiation of SGLT2 inhibitors is important, irrespective of ejection fraction: SOLOIST‐WHF in perspective. ESC Heart Fail.

[5] Lee SY, Lee TW, Park GT (2021). Sodium/glucose co-transporter 2 inhibitor, empagliflozin, alleviated transient expression of SGLT2 after myocardial infarction. Korean Circ J.

[6] Liu Y, Wu M, Xu J, Xu B, Kang L (2021). Empagliflozin prevents from early cardiac injury post myocardial infarction in non-diabetic mice. Eur J Pharm Sci.

[7] McMurray JJ, Solomon SD, Inzucchi SE (2019). Dapagliflozin in patients with heart failure and reduced ejection fraction. N Engl J Med.

[8] Anker SD, Butler J, Filippatos G (2021). Empagliflozin in heart failure with a preserved ejection fraction. N Engl J Med.

[9] Butler J, Jones WS, Udell JA (2024). Empagliflozin after acute myocardial infarction. N Engl J Med.

[10] Chang TY, Lu CT, Huang HL (2022). Association of sodium-glucose cotransporter 2 (SGLT2) inhibitor use with cardiovascular and renal outcomes in type 2 diabetes mellitus patients with stabilized acute myocardial infarction: a propensity score matching study. Front Cardiovasc Med.

[11] James S, Erlinge D, Storey RF (2024). Dapagliflozin in myocardial infarction without diabetes or heart failure. NEJM Evid.

[12] Kwon O, Myong JP, Lee Y (2023). Sodium-glucose cotransporter-2 inhibitors after acute myocardial infarction in patients with type 2 diabetes: a population-based investigation. J Am Heart Assoc.

[13] von Lewinski D, Kolesnik E, Tripolt NJ (2022). Empagliflozin in acute myocardial infarction: the EMMY trial. Eur Heart J.

[14] Lyu YS, Oh S, Kim JH, Kim SY, Jeong MH (2023). Comparison of SGLT2 inhibitors with DPP-4 inhibitors combined with metformin in patients with acute myocardial infarction and diabetes mellitus. Cardiovasc Diabetol.

[15] Mao L, Cai D, Chi B (2023). Dapagliflozin reduces risk of heart failure rehospitalization in diabetic acute myocardial infarction patients: a propensity score-matched analysis. Eur J Clin Pharmacol.

[16] Paolisso P, Bergamaschi L, Gragnano F (2023). Outcomes in diabetic patients treated with SGLT2-Inhibitors with acute myocardial infarction undergoing PCI: the SGLT2-I AMI PROTECT Registry. Pharmacol Res.

[17] Peters SA, Colantonio LD, Dai Y (2021). Trends in recurrent coronary heart disease after myocardial infarction among US women and men between 2008 and 2017. Circulation.

[18] Sinha T, Khilji F, Laraib F (2024). The effectiveness of sodium-glucose cotransporter-2 (SGLT2) inhibitors on cardiovascular outcomes and all-cause mortality in patients with acute coronary syndrome: a systematic review and meta-analysis. Cureus.

[19] Griffin M, Rao VS, Ivey-Miranda J (2020). Empagliflozin in heart failure: diuretic and cardiorenal effects. Circulation.

[20] Batzias K, Antonopoulos AS, Oikonomou E (2018). Effects of newer antidiabetic drugs on endothelial function and arterial stiffness: a systematic review and meta-analysis. J Diabetes Res.

[21] Oshima H, Miki T, Kuno A (2019). Empagliflozin, an SGLT2 inhibitor, reduced the mortality rate after acute myocardial infarction with modification of cardiac metabolomes and antioxidants in diabetic rats. J Pharmacol Exp Ther.

[22] Lahnwong C, Palee S, Apaijai N (2020). Acute dapagliflozin administration exerts cardioprotective effects in rats with cardiac ischemia/reperfusion injury. Cardiovasc Diabetol.

[23] Li Z, Li A, Sun D, Shu Y (2024). Effect of SGLT-2 inhibitors on prognosis in diabetic patients with acute myocardial infarction: a systematic review and meta-analysis. Rev Cardiovasc Med.

[24] Zannad F, Ferreira JP, Pocock SJ (2020). SGLT2 inhibitors in patients with heart failure with reduced ejection fraction: a meta-analysis of the EMPEROR-Reduced and DAPA-HF trials. Lancet.

[25] Chew DS, Heikki H, Schmidt G (2018). Change in left ventricular ejection fraction following first myocardial infarction and outcome. JACC Clin Electrophysiol.

